# Supplementary material for: 2D effects enhance precision of gradient-based tissue patterning
Source: iScience. 2023 Sep 16;26(10):107880. doi: 10.1016/j.isci.2023.107880 (PMC10550716; doi:10.1016/j.isci.2023.107880)
Supplement: Document S1. Figures S1–S4 [file mmc1.pdf]

**iScience, Volume *26***

## Supplemental information

# 2D effects enhance precision of gradient-based tissue patterning

**Yuchong Long, Roman Vetter, and Dagmar Iber**

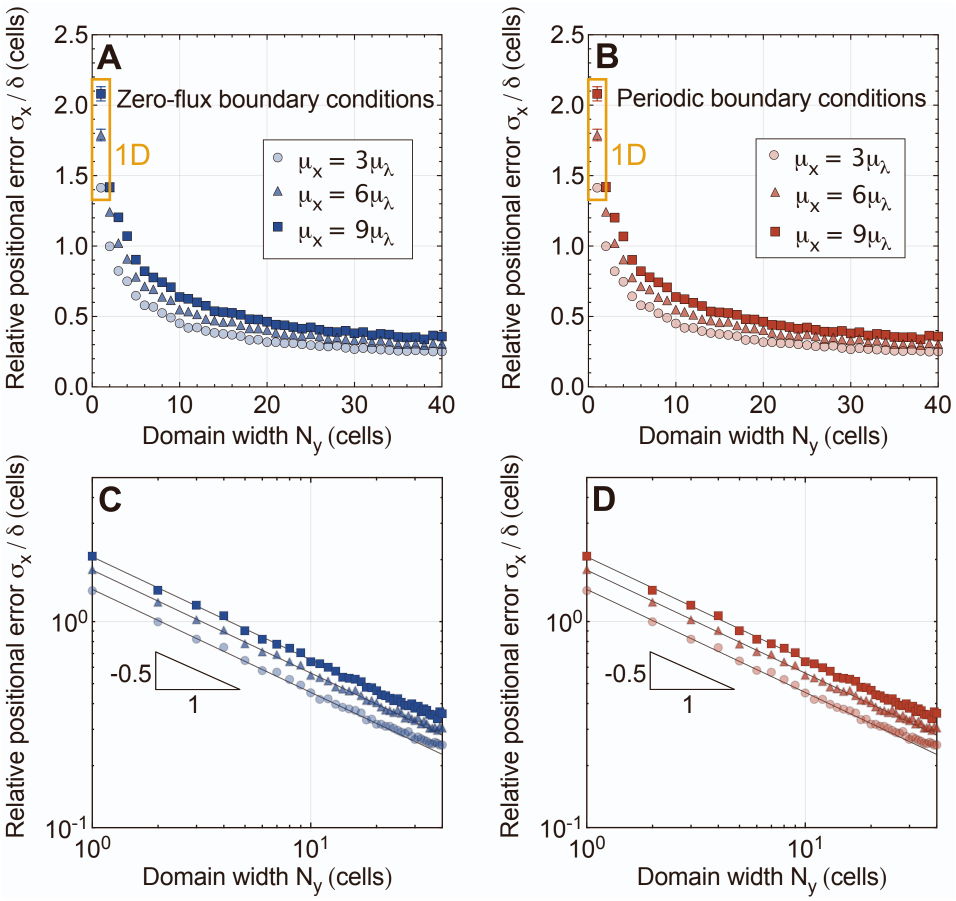


**Figure S1: Impact of domain width on gradient readout precision for small cell diameters, related to Figure 2.** Simulations analogous to those shown in Fig. 2C,D,G,H, except that the cell diameter is halved to $\delta = 2.5\,\mu\text{m}$. See caption of Fig. 2 for details.

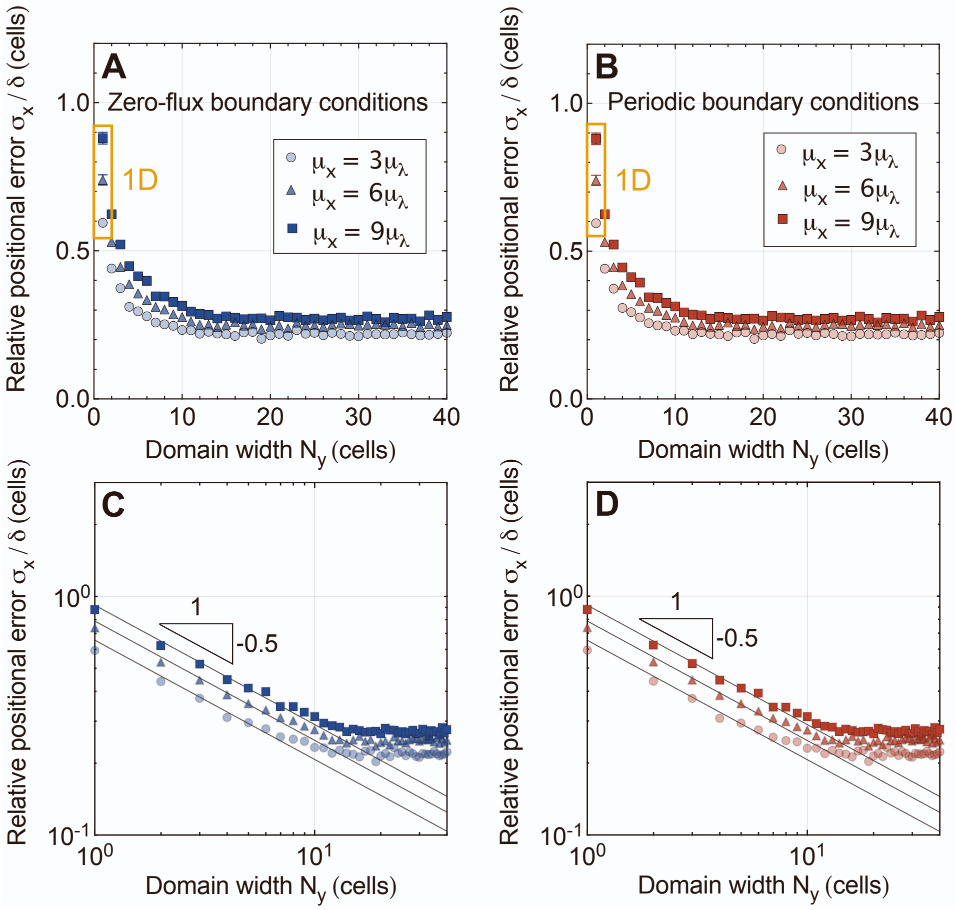


**Figure S2: Impact of domain width on gradient readout precision for large cell diameters, related to Figure 2.** Simulations analogous to those shown in Fig. 2C,D,G,H, except that the cell diameter is increased to $\delta = 12.5\,\mu\text{m}$. See caption of Fig. 2 for details.

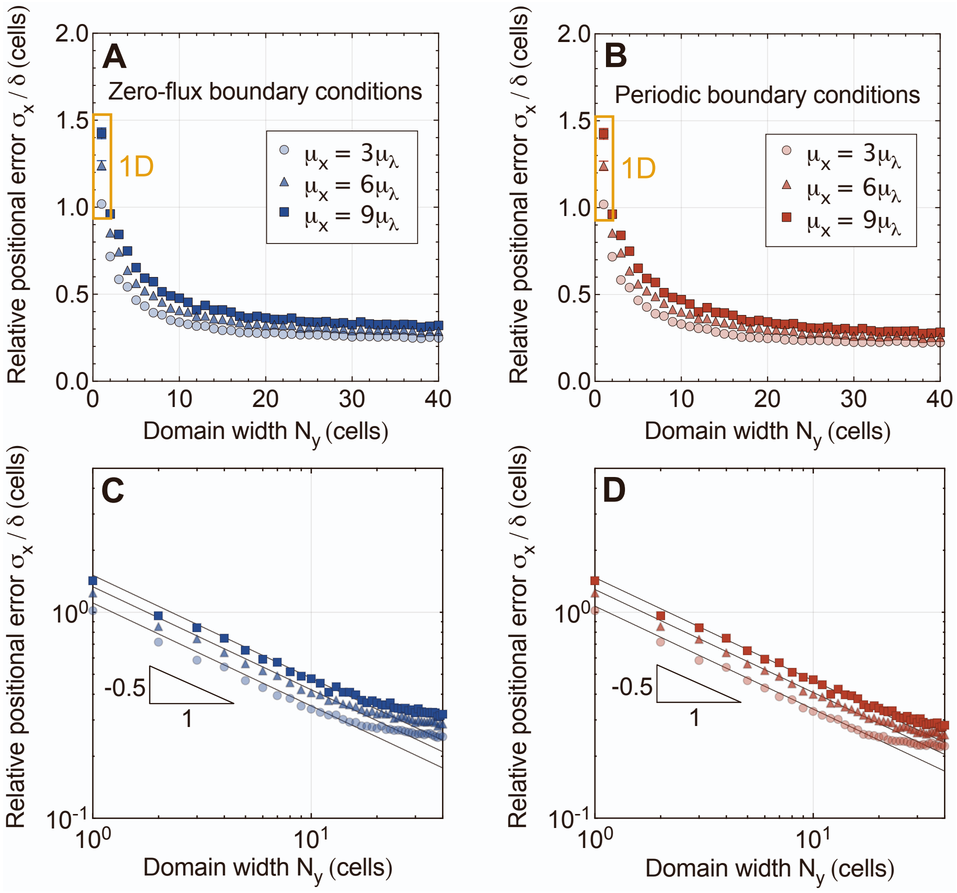


**Figure S3: Impact of domain width on gradient readout precision averaged across the domain width, related to Figure 2.** Simulations analogous to those shown in Fig. 2C,D,G,H, except that the positional error averaged over all cell rows is plotted. See caption of Fig. 2 for details.

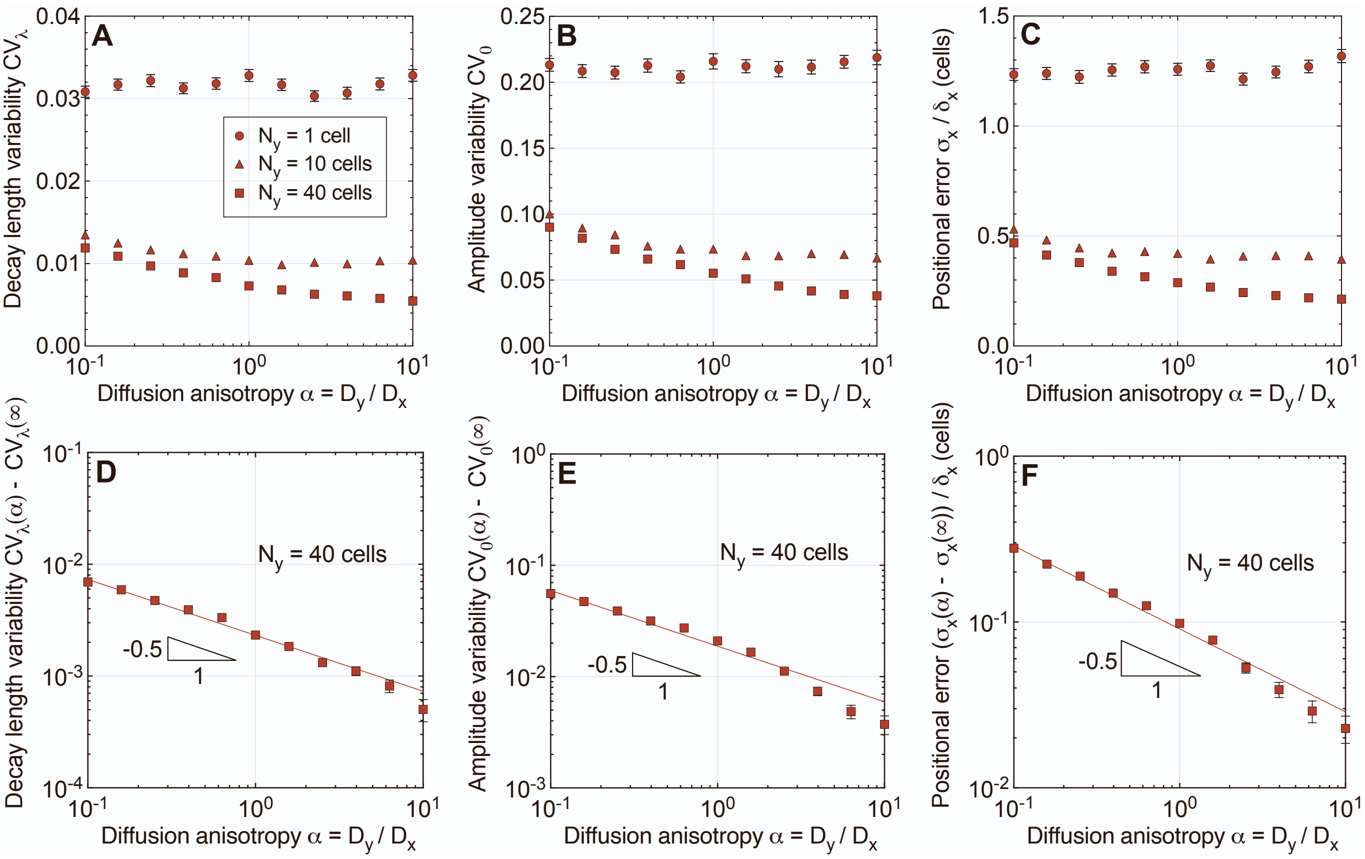


**Figure S4: Effect of anisotropic morphogen transport on patterning precision, related to Figure 2 and Section "Fast transverse diffusion increases patterning precision in wide tissues". A,B** Gradient variability as a function of the degree of orthotropy in morphogen transport, $\alpha = D_y/D_x$. **C** Positional error at $\mu_x = 6\mu_\lambda$ as a function of $\alpha$. **D–F** Log-log plots of A–C at a tissue width of $N_y = 40$ cells, revealing square-root scaling when $N_y$ is large enough. Data points indicate mean ± SEM from $n = 1000$ independent 2D gradients obtained on a tissue of square cells ($\delta_x = \delta_y$).
